# Giant Mesenteric Hemangiolymphangioma Enveloping the Small Bowel: A Case Report

**DOI:** 10.7759/cureus.97740

**Published:** 2025-11-25

**Authors:** Yufei Wang, Hui Luo

**Affiliations:** 1 Department of Anorectal Surgery, The Affiliated Traditional Chinese Medicine Hospital of Southwest Medical University, Luzhou, CHN; 2 Department of Operations Management, Luzhou Traditional Chinese Medicine Hospital, Luzhou, CHN

**Keywords:** hemangiolymphangioma, lymphangioma, mesentery, radical resection, small bowel

## Abstract

Hemangiolymphangioma is a rare type of lymphangioma marked by a mixture of blood vessels and lymphatic vessels, usually found in infants and often on the surface of the body. This article discusses a case of a giant hemangiolymphangioma on the mesentery of the small intestine in a 34-year-old man, where a preoperative CT scan showed a mass in the mesentery. The patient was successfully treated with radical resection, and six months after surgery, he had no specific discomfort, and a follow-up abdominal CT showed no signs of recurrence. This case suggests that for localized mesenteric hemangiolymphangioma, complete surgical removal is a safe and effective treatment. This case provides important insights for managing giant mesenteric hemangiolymphangioma and highlights how a surgery-focused approach can really improve patients' quality of life.

## Introduction

Hemangiolymphangioma is an extremely rare benign tumor that originates from mesenchymal tissue, with its formation mechanism still unclear, which may be caused by developmental defects or issues with how blood and lymphatic vessels form. Although very rare, trauma and surgery can also damage the vascular and lymphatic systems, thereby leading to the formation of hemangiolymphangioma. These tumors can happen at any age, but they're most common in infants and pretty rare in adults. The incidence of hemolymphangioma is 1.2% to 2.8% in neonates, and approximately 90% of the patients are diagnosed under the age of 2 years. They are often located on the surface of the body and can show up anywhere on the body [1]. Less than 5% of all hemangiolymphangiomas are found in the abdomen [2]. Some common places where these tumors are found include the head and neck, spleen, pancreas, esophagus, stomach, small intestine, colon, and rectum, and those found in the mesentery of the small intestine are even rarer. This article shares a case of a giant hemangiolymphangioma on the small intestine's mesentery that was successfully treated with radical resection.

## Case presentation

**Figure 1:**
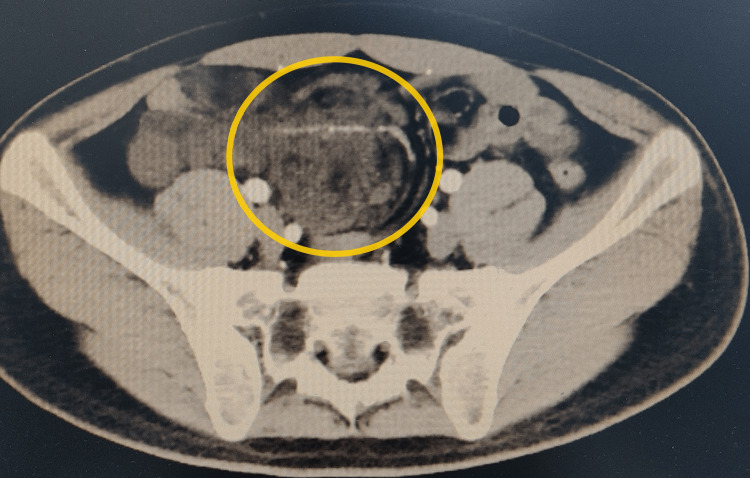
Axial CT scan of the abdomen The yellow circle indicates the mesenteric mass. The abdominal CT scan indicates a mass in the mesentery of the small intestine, measuring approximately 114*62*127mm, showing visible mesenteric blood vessels and slightly enlarged lymph nodes.

A 34-year-old male patient presented with abdominal pain and weight loss, but no other symptoms. The patient was initially seen at a local hospital and then referred to our department for inpatient treatment. An enhanced abdominal CT scan indicated a mass in the mesentery of the small intestine, measuring approximately 114*62*127mm, showing visible mesenteric blood vessels and slightly enlarged lymph nodes, which suggested possible mesenteric torsion (Figure 1). The patient's complete blood count, routine stool and urine tests, biochemistry, coagulation, and CEA and AFP test results showed no significant abnormalities.

**Figure 2:**
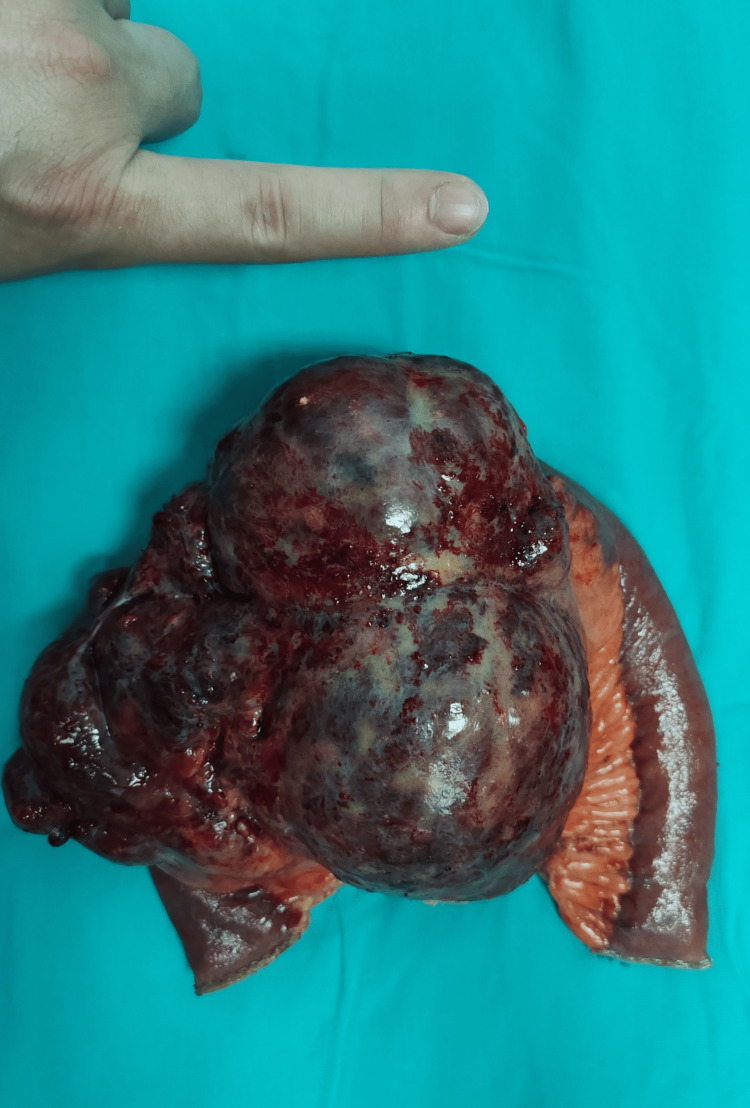
The resected small intestine and mesentery. A mesenteric hemangiolymphangioma surrounding the small intestine, measuring approximately 15*14.5*10cm.

**Figure 3:**
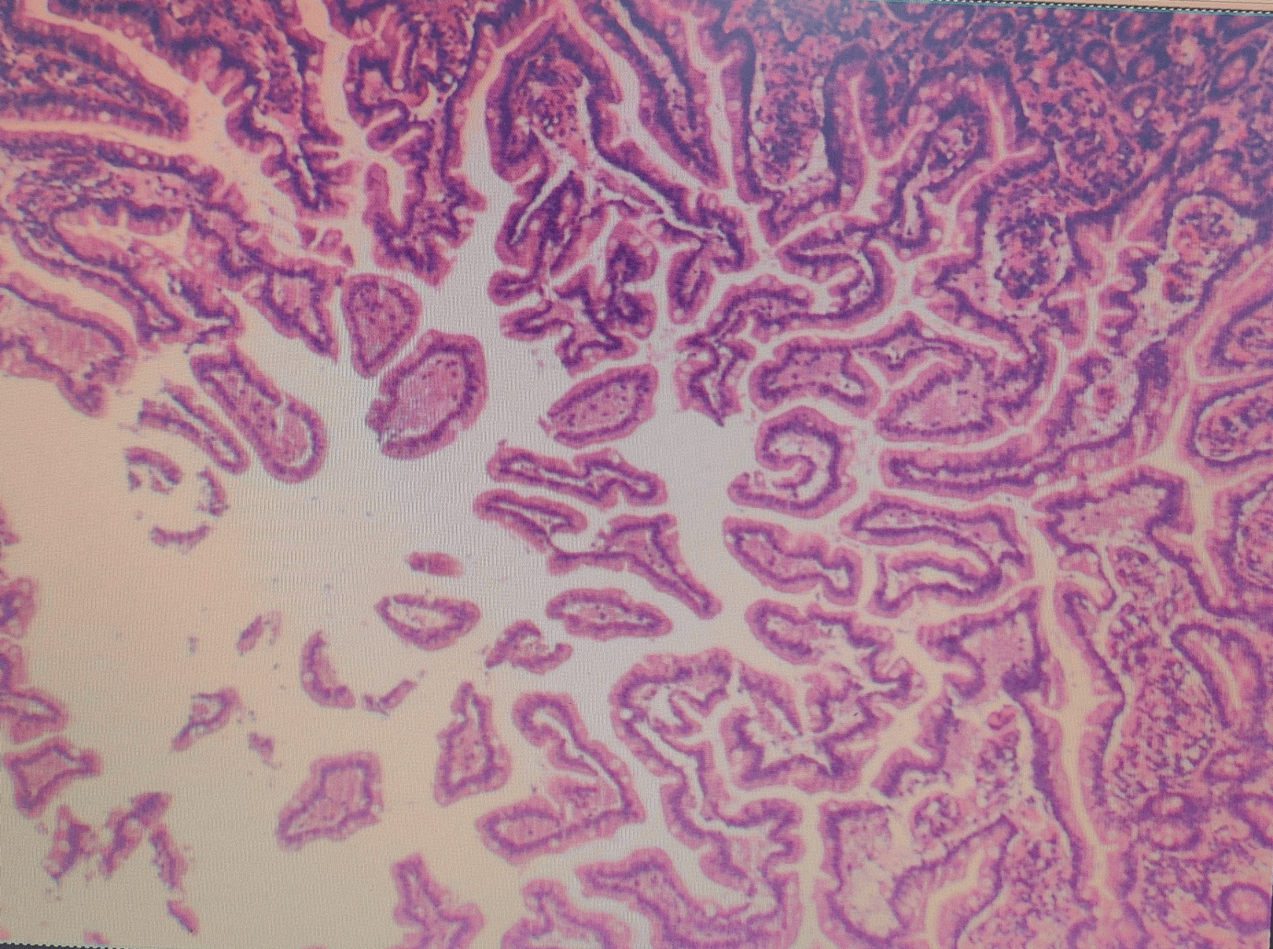
The postoperative pathology confirmed hemangiolymphangioma Histopathological examination reveals proliferative and dilated blood vessels and lymphatic channels.

Due to the large abdominal tumor, the patient underwent surgery for resection. During the operation, we found a large mass coming from the mesentery of the small intestine, affecting part of the small intestine, with a 360-degree twist of the mesentery and some dilated and swollen intestinal segments (Figure 2). Microscopic examination revealed dilated and proliferative blood vessels and lymphatic vessels within the mesenteric mass. Immunohistochemistry tests showed the tumor cells were positive for CD34(+), CD31(+), D2-40(+), and Ki67 (about 5%), and negative for CK(-). The pathology results confirmed it was a hemangiolymphangioma based on the immunohistochemistry findings (Figure 3).

## Discussion

Hemangiolymphangiomas are clinically rare, mostly congenital, and the mechanism of formation is not yet clear. They may result from congenital malformations of blood vessels and lymphatic vessels; trauma and surgery may lead to damage to the vascular and lymphatic systems, which can lead to the formation of vascular lymphangiomas, though this is very uncommon. Traditionally, lymphangiomas are classified into four types: capillary lymphangioma, cavernous lymphangioma, cystic lymphangioma, and vascular lymphangioma [3]. Vascular lymphangioma is categorized as a type of lymphangioma, but it is now more commonly considered a mixed vascular and lymphatic tumor, also known as a hemangiolymphangioma, a type of mixed tumor that is clinically less common than simple lymphangiomas. This condition is more common in kids, often congenital, occurring as single or multiple growths, ranging from a few millimeters to a few centimeters in diameter. The incidence of hemangiolymphangioma in newborns is between 1.2% and 2.8%, with about 90% of patients diagnosed before the age of two [4]. They are more commonly found in the head and neck region [5], and there have also been cases in the pancreas and spleen [6,7], while they are pretty rare in the gastrointestinal tract [8], and those in the mesentery of the small intestine are even less common.

Mesenteric hemangiolymphangioma in the small intestine is quite rare clinically, with common clinical manifestations including abdominal mass, abdominal pain, blood in stool, and intestinal obstruction, which are often difficult to differentiate from other diseases of the small intestine, making preoperative and intraoperative diagnosis challenging. Abdominal hemangiolymphangioma can grow fast and might exert a mass effect on adjacent visceral organs, mainly the bowel loops. Patients with large abdominal hemangiolymphangioma can present with abdominal discomfort, acute abdominal pain, and bowel obstruction. Melena, or anemia, could be a symptom if the mass infiltrates the bowel mucosa. Rupture and infection can also occur. Preoperative diagnosis mainly relies on imaging: (1) A plain CT scan with contrast can be quite useful, primarily depending on the vascular proportion within the tumor; for tumors with a higher proportion of blood vessels, enhanced scans reveal increased visibility, particularly more evident in the venous and delayed phases; for those with a lower vascular proportion, enhancement is not obvious, which can lead to misdiagnosis. In this case, the enhanced scan showed progressive enhancement of the tumor, more pronounced in the venous phase; (2) MRI examination of cystic fluid signals depends on the proportion of blood vessels and lymphatic vessels within the tumor, where T1W1 signals typically show low or slightly low intensity, and T2W1 signals predominantly show high intensity. Intraoperative anatomy can be important for diagnosis. Hemangiolymphangioma typically has a softer texture, clear boundaries, and shows internal multilocular changes, with cystic fluid containing blood and lymphatic fluid. Hemangiolymphangioma is characterized by collections of mucosal lymphatic vessels and blood vessels. The definitive diagnosis of hemangiolymphangioma mainly relies on postoperative pathology, where many dilated blood and lymphatic vessels are visible under the microscope; immunohistochemistry can help further differentiate, as both vascular endothelial cells and lymphatic endothelial cells express CD31 and CD34, while D2-40 is only expressed in lymphangiomas and some malignant vascular tumors, making it relatively easy to confirm the diagnosis using these indicators. In this case, tumor cells expressed CD34 (+) and D2-40 (+), confirming the pathological diagnosis of hemangiolymphangioma.

Hemangiolymphangiomas typically occur before the age of 2, generally grow slowly and can remain asymptomatic for a long time. However, sometimes their growth can suddenly accelerate, putting pressure on nearby tissues or other organs [9]. The patient is 34 years old and might have had this condition for years without any symptoms. He went to the hospital because of a recent increase in the size of an abdominal mass and symptoms like abdominal pain and weight loss and was admitted for tests. After getting a clear diagnosis, they had surgery to remove it, and the surgical margins were clean. There's been no recurrence six months after the surgery.

Hemangiolymphangioma, although a benign tumor, has an aggressive growth behavior [9,10]. The tumor can compress the intestines and surrounding solid organs, leading to symptoms of acute abdomen like abdominal pain, bloating, intestinal obstruction, and volvulus. When some tissues and organs get pushed out of place due to compression, it can affect normal physiological functions; the tumor itself may also become infected, bleed, or rupture. So, it's important to treat it quickly once it's found. Conservative treatments such as puncture aspiration and injection of sclerosing agents have poor efficacy and a high recurrence rate, which makes complete surgical removal the best option. However, even after complete resection, the recurrence rate is still 10%-27% [11]. Although the tumor's location and size vary, completely removing it is key to helping patients feel better and stopping it from coming back. Other treatment options include laser therapy, cryotherapy, and sclerosing agent injections, but they have a higher recurrence rate compared to surgical resection. It is important to note that the lesion tissue of hemangiolymphangioma is accompanied by vascular proliferation, and the depth of how far it has spread needs to be checked before surgery to prevent uncontrollable bleeding and perforation.

The surgical method should be chosen based on the size, location, and surrounding invasion of the tumor, with the goal of complete resection without damaging surrounding tissues and organs. If the tumor is tightly stuck to nearby structures and cannot be separated, it should be taken out along with part or all of the affected organ, and the surrounding lymphatic vessels should be tied off. If the tumor involves major blood vessels, the surgery has to be done very carefully. For extensive and multiple abdominal cavity tumors, it's important to explore carefully to make sure nothing is missed. You need to be really detailed during the operation to prevent tumor rupture. If the tumor is large and affects the operation, you can drain the fluid before taking it out completely. Laparoscopic surgery works well for patients with small, localized tumors that aren't stuck to nearby tissues. Generally, the prognosis is good after complete surgical resection, with rare recurrence, and it's a good idea to have regular check-ups with ultrasound or CT. Therefore, to keep an eye out for any possible recurrence or spread, you'll need regular follow-ups after surgery for this condition.

## Conclusions

This case report successfully demonstrates the complete surgical resection and treatment process of a hemangiolymphangioma of the mesentery in the small intestine. Postoperative pathology confirmed it to be a hemangiolymphangioma, and the patient recovered well without complications, and during short-term follow-up, there were no signs of recurrence. This case suggests that for localized mesenteric hemangiolymphangioma, complete surgical resection is a safe and effective way to treat it. Thorough preoperative imaging is key to understanding the extent of the lesion and planning the surgery. We still need longer follow-up in the future to further assess its long-term efficacy and to accumulate more cases to better understand treatment strategies for this rare disease.
